# Huntington’s Disease: A Review of the Known PET Imaging Biomarkers and Targeting Radiotracers

**DOI:** 10.3390/molecules25030482

**Published:** 2020-01-23

**Authors:** Klaudia Cybulska, Lars Perk, Jan Booij, Peter Laverman, Mark Rijpkema

**Affiliations:** 1 Department of Radiology and Nuclear Medicine, Radboud University Medical Center, Geert Grooteplein-Zuid 10, 6525 EZ Nijmegen, The Netherlands; jan.booij@radboudumc.nl (J.B.); peter.laverman@radboudumc.nl (P.L.); mark.rijpkema@radboudumc.nl (M.R.); 2 Radboud Translational Medicine B.V., Radboud University Medical Center, Geert Grooteplein 21 (route 142), 6525 EZ Nijmegen, The Netherlands; lars.perk@radboudumc.nl; 3 Department of Radiology and Nuclear Medicine, Amsterdam University Medical Centers, Academic Medical Center, Meibergdreef 9, 1105 AZ Amsterdam, The Netherlands

**Keywords:** Huntington’s disease, mutant huntingtin, Positron Emission Tomography, radiochemistry, fluorine-18, carbon-11, radiopharmaceuticals, [^11^C]raclopride, [^18^F]MNI-659

## Abstract

Huntington’s disease (HD) is a fatal neurodegenerative disease caused by a CAG expansion mutation in the *huntingtin* gene. As a result, intranuclear inclusions of mutant huntingtin protein are formed, which damage striatal medium spiny neurons (MSNs). A review of Positron Emission Tomography (PET) studies relating to HD was performed, including clinical and preclinical data. PET is a powerful tool for visualisation of the HD pathology by non-invasive imaging of specific radiopharmaceuticals, which provide a detailed molecular snapshot of complex mechanistic pathways within the brain. Nowadays, radiochemists are equipped with an impressive arsenal of radioligands to accurately recognise particular receptors of interest. These include key biomarkers of HD: adenosine, cannabinoid, dopaminergic and glutamateric receptors, microglial activation, phosphodiesterase 10 A and synaptic vesicle proteins. This review aims to provide a radiochemical picture of the recent developments in the field of HD PET, with significant attention devoted to radiosynthetic routes towards the tracers relevant to this disease.

## 1. Introduction

The purpose of this review is to provide a radiochemistry focused summary of the recent advancements in Positron Emission Tomography (PET) imaging of a rare genetic condition, Huntington’s disease (HD). The prevalence of HD is 5 to 10 cases per 100,000 people worldwide [1]. It progresses with fatal and devastating psychiatric, cognitive and motor impairments, caused by mutant huntingtin (mHTT) protein expression. Some excellent reviews on the molecular imaging of HD have been published, however focusing primarily on clinical data [2,3,4,5]. This review is divided according to HD biomarkers thought to be most affected by the pathology. For each biomarker, a suitable PET tracer and its radiosynthesis are provided, together with the most important PET data, both clinical and preclinical, if available. The authors’ intention is to highlight the importance of radiochemistry and design of novel and highly specific radioligands for PET imaging, which will further our understanding of the changes orchestrated by mutant huntingtin and may eventually lead to the invention of disease-modifying treatments.

### 1.1. Positron Emission Tomography

PET plays a pivotal part in the diagnosis and understanding of neurological pathophysiologies. It is a non-invasive molecular imaging technique employing radiopharmaceuticals, which, after crossing the blood–brain barrier, bind to a specific molecular target, such as a transporter or receptor, and enable accurate tracking of changes in their function. Nowadays, PET is equipped with an array of radiolabelled biomarkers for neuroimaging in psychiatry and neurodegenerative pathologies, such as Parkinson’s disease (PD), Alzheimer’s disease (AD) and HD.

### 1.2. Huntington’s Disease

Although considerable progress has been made towards identifying some of the mechanisms involved in the HD pathogenesis, there are currently no disease-modifying strategies available [6]. The uncovering of the huntingtin (*htt*) mutation in 1993 has enabled intensified research efforts with the hope to slow down or stop progressive neuronal damage [4]. HD is an autosomal dominant disease of the central nervous system (CNS) caused by an expansion of the CAG sequence in the *huntingtin* gene (*HTT*), located on chromosome 4 [3,7]. As a result, the expressed huntingtin protein is a mutant (mHTT) with an expanded polyglutamine tract. It has been proposed that a flawed proteostasis network results from the aggregation of mHTT, initiating a cascade of devastating consequences for synchronised neuroreceptors [8]. Individuals with 7-12 CAG tracts are usually considered healthy, whilst those with 35 suffer from HD [4]. Additionally, full penetrance and consequently, rapid progression of the disease, is associated with more than 40 repeats [9]. There is an inverse correlation between the number of CAG repeats and the age of HD onset, usually mid-life, but juvenile forms have also been reported [10,11]. Aggregated mHTT, also called inclusion bodies (IBs), manifests itself primarily in a brain region called the striatum, where it causes dysfunction of medium spiny neurons (MSNs) and eventually their death [8]. HD-affected subjects suffer from different types of impairments: motor (chorea, loss of coordination and involuntary movements), cognitive, in the form of widely understood dementia, and psychiatric (depression, anxiety and personality changes).

## 2. Indicators of Huntington’s Disease: From A to Z

Within the complex brain networks, several molecular mechanisms have been recognised for their involvement in the HD pathogenesis. These include glucose metabolism, dopaminergic system, phosphodiesterases and neuroinflammation. PET imaging of these biomarkers provides useful insight into the disease progression by quantifying, for example, receptor expression and density. To the best of our knowledge, no PET tracer targeting mHTT has been proposed to date. The emergence of such a radioligand could aid validation of novel disease-modifying therapies aimed at lowering levels of this neurotoxic aggregate. This review summarises the key biochemical targets within the central nervous system which could be relevant for HD and the corresponding PET radioligands, along with their radiosynthetic routes.

### 2.1. Adenosine Receptors

Somewhat less studied in the context of HD, adenosine receptors have been considered potential biomarkers of the pathology due to their involvement in neurotransmission [12,13]. Adenosine is a vital dopaminergic and glutamatergic modulator [14]. It acts as an inhibitory neurotransmitter by monitoring energy levels and usage. It exerts its action through four G protein-coupled receptors, A_1_, A_2A_, A_2B_ and A_3_. A_1_ receptors are expressed on dynorphinergic MSNs which also co-express dopamine D_1_ receptors (for a more detailed explanation of D_1_ receptor involvement in HD, see Section 2.3). Enkephalinergic MSNs co-express A_2A_ and dopamine D_2_ receptors. The latter are found abundantly in the striatum and significantly less in other brain regions [15,16,17]. Both types have been investigated, although not considerably in HD, with PET imaging in preclinical and clinical settings.

#### 2.1.1. [^18^F]CPFPX

**Scheme 1:**
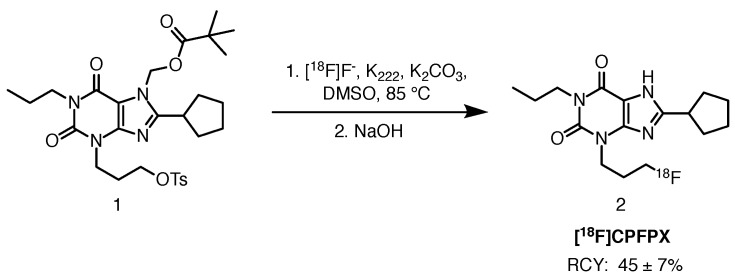
Radiolabelling of [^18^F]CPFPX, starting from the tosyloxy precursor **3** [18].

[^18^F]CPFPX is a potent xanthine-based antagonist of the adenosine A_1_ receptor. Its binding selectivity over A_2A_ is 1200-fold [18]. The radioligand is synthesised via nucleophilic aliphatic substitution at the tosylate leaving group in the presence of Kryptofix 222 and potassium carbonate, followed by deprotection of the pivaloyloxymethyl group with an aqueous solution of sodium hydroxide (Scheme 1). The average radiochemical yield (RCY) is 45 ± 7%, with molar activity values exceeding 270 GBq/μmol. Molar activity expresses the measured radioactivity per mole of compound and is commonly reported in GBq/μmol [19]. Radiochemical purity (RCP) exceeded 98%. In vivo rodent experiments by Holschbach et al. and later by Bauer and co-workers, confirmed the suitability of the tracer for A_1_ receptor studies, owing to favourable kinetics and behaviour in the presence of a standard A_1_ desfluorinated antagonist, DPCPX [18,20].

The tracer was used by Matusch et al. in premanifest and manifest HD individuals in an effort to elucidate the role of adenosine A_1_ receptors in the pathology [21]. The former group was divided into far and near, based on the number of years until the calculated clinical onset. It was discovered that [^18^F]CPFPX binding was globally higher for the far premanifest subjects than for the healthy controls, then levelling up for the near-to-onset subset and finally, decreasing in the caudate nucleus (part of the striatum), frontal cortex and amygdala for the symptomatic cohort. These results, although preliminary, suggest potential discriminatory power of adenosine A_1_ biomarker for HD phenoconversion.

#### 2.1.2. [^11^C]KF18446

**Scheme 2:**
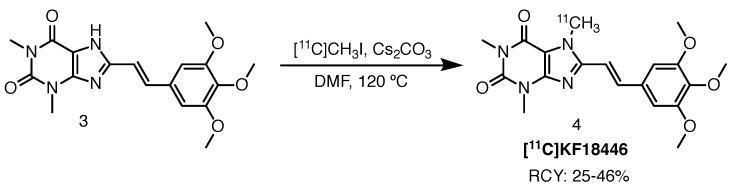
Radiolabelling of [^11^C]KF18446, starting from the desmethyl precursor **3** [23].

Ishiwata et al. investigated the adequacy of adenosine A_2A_ receptor imaging with [^11^C]KF18446, another xanthine-based ligand, based on evidence that there is a marked reduction in the receptor density of HD patients, particularly in the caudate nucleus, putamen and external globus pallidus [22,23]. Like the caudate nucleus, putamen is part of the striatum and together, the three brain areas are part of the so-called basal ganglia—responsible for the coordination of motor functioning. The study was performed with the aforementioned radioligand due to its promising imaging properties. The tracer was accessed through ^11^C-methylation with [^11^C]methyl iodide ([^11^C]CH_3_I) in the presence of caesium carbonate in DMF at 120 ${}^{\circ }$C (Scheme 2). Radiochemical yields ranged between 25% and 46% with molar activities of 10–72 GBq/μmol. Radiochemical purity (RCP) was higher than 99%.

In 2002, Ishiwata and colleagues published an interesting PET study in which rats had been injected with quinolinic acid, an excitotoxin, to mimic HD pathology [23]. The compound causes damage to the dopaminergic neurons in the striatum. PET data, co-registered with Magnetic Resonance Imaging (MRI), were accompanied by in vitro and ex vivo autoradiography. The images displayed clear degeneration of not only A_2A_, but also D_1_ and D_2_ receptors, calculated as a difference in the binding of the tracer between the non-lesioned and lesioned sides [23].

### 2.2. Cannabinoid Receptors

Gaining better understanding of the implication of the endocannabinoid system in synaptic transmission could bring us closer to unravelling the mechanism of HD and perhaps, further down the line, designing an efficient therapeutic against it. Cannabinoid receptor type 1, CB_1_, is one of the major cannabinoid receptors, belonging to the G protein-coupled family, expressed abundantly in striatal MSNs, and overlooking the complex process of inhibitory neurotransmission [3,24]. The marked loss of CB_1_ receptors in post mortem human brains, described by Glass et al., was one of the first receptor pathologies documented in HD [25]. It was most pronounced in the caudate nucleus, putamen and globus pallidus externus, even in the very early stage of the disease [26].

#### 2.2.1. ^18^F]MK-9470

PET allows for non-invasive in vivo imaging of various CNS receptors, including CB_1_. Van Laere and colleagues administered a selective inverse agonist, [^18^F]MK-9470, to 20 manifest HD patients [27]. Loss of PET signal was detected in the caudate and putamen, as well as the grey matter of cerebrum, brain stem and cerebellum. The HD cohort showed a significant loss of cannabinoid CB_1_ receptors compared to the healthy controls. No relationship was elucidated between receptor binding and clinical HD scales or the number of CAG repeats. With this investigation, the researchers pioneered the use of CB_1_ as a biomarker for HD PET imaging.

**Scheme 3:**
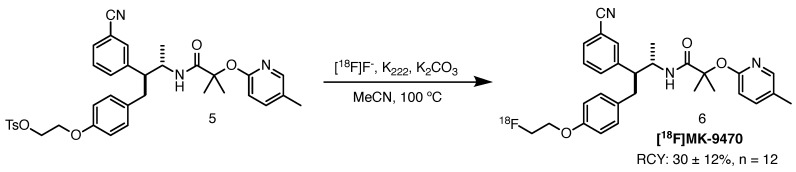
Radiolabelling of [^18^C]MK-9470, starting from the tosyloxy precursor **5** [28].

[^18^F]MK-9470 can be radiolabelled by aliphatic nucleophilic substitution with [^18^F]F^−^ using the corresponding tosyloxy precursor. Thomae and co-workers published an automated Good Manufacturing Practice (GMP) compliant radiosynthetic route towards this ligand (Scheme 3) [28]. The authors reported an average RCY of 30 ± 12% (*n* = 12), RCP and molar activity exceeding 95% and 370 GBq/μmol, respectively.

### 2.3. Dopaminergic Receptors

Dopaminergic receptors D_1_ and D_2_ constitute another part of the complex neurotransmission system which is affected by mHTT, mainly postsynaptically. Dysfunction of and fluctuations in the availability of these receptors have been one of the hallmarks of HD. Approximately half of MSNs express D_1_ implicated in the ”direct pathway”, while the other half expresses mainly D_2_ receptors implicated in the ”indirect pathway” [29]. Postsynaptic nerve terminals which express D_1_ and D_2_ receptors, are severely affected by MSN degradation, caused by the presence of neurotoxic mHTT. PET radioligands which visualise density changes of these receptors can also provide insight into the pathology.

#### 2.3.1. [^11^C]SCH-23390

SCH-23390 is a potent halobenzazepine-based D_1_ receptor antagonist [30,31]. The active form of this molecule is the *R*-enantiomer. Interestingly, in 1985, Friedman et al. began their evaluation of a ^76^Br-brominated version of the molecule using small animal PET [31]. In 1989, DeJesus and colleagues evaluated the carbon-11 version of the ligand, which contains a ^11^C-tagged *N*-methyl moiety, as a potential tracer for the imaging of CNS D_1_ levels [32]. Nowadays, [^11^C]SCH-23390 is still frequently used in preclinical and clinical PET studies of the D_1_ receptor [33].

**Scheme 4:**
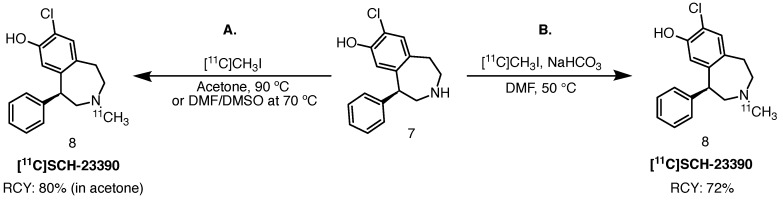
**A.** Radiolabelling of [^11^C]SCH-23390, starting from the desmethyl precursor **7**, described by Halldin and colleagues [35]; **B.** Method from DeJesus and colleagues [34].

Typical radiolabelling via ^11^C-alkylation of the secondary amine is achieved with [^11^C]methyl iodide in the presence of a mild base, such as sodium bicarbonate, in DMF at 50 ${}^{\circ }$C [34]. DeJesus et al. described the wet method, starting with lithium aluminium hydride-mediated reduction of cyclotron-acquired [^11^C]CO_2_, followed by iodination with hydroiodic acid to yield [^11^C]methyl iodide. Radiolabelling was achieved by bubbling the resulting gaseous ^11^C-methylating agent into a solution of the corresponding amine precursor and sodium hydrogen carbonate (Scheme 4). The authors reported RCY of 72% over 30 runs (based on [^11^C]methyl iodide). The product obtained was radiochemically pure; however, contaminated with approximately 5% of the desmethyl precursor. Molar activities ranged from 370 to 8695 GBq/mmol. Halldin et al. also achieved successful radiolabelling of [^11^C]SCH-23390, starting from the free base desmethyl precursor in acetone or DMSO-DMF at elevated temperatures [35]. Radiolabelling in acetone yielded the desired tracer in 80% RCY (based on [^11^C]methyl iodide) and RCP higher than 99%. Publications that followed thereafter described radiolabelling of this tracer based on either of the protocols.

Sedvall and co-workers investigated the performance of the radioligand in HD-affected individuals [36]. Five clinically diagnosed patients with motor dysfunction and one asymptomatic gene carrier were chosen alongside five healthy male volunteers. The authors reported a 50% decrease in D_1_ receptor density in the putamen in comparison to the healthy subjects. For the sole asymptomatic HD individual, this value lied in the lower boundary of that of the healthy ones. Andrews et al. studied the rate of dopamine D_1_ and D_2_ receptor loss over 40 months in a larger group including 9 asymptomatic, 4 symptomatic and 3 patients at risk, complemented by 7 healthy controls [37]. In the first group [^11^C]SCH-23390 signal was lost at a mean rate of 2% annually, with some patients progressing actively with a mean yearly loss of 4.5%. For the manifest individuals, a mean annual decrease of radioligand binding of 5.0% was reported.

#### 2.3.2. [^11^C]NNC-112

In the aforementioned publication, the study of longitudinal D_1_ receptor changes was performed with a carbon-11 based ligand, [^11^C]NNC-112 [38]. It is a derivative of [^11^C]SCH-22390 with a benzofuran substituent in the 5-position of the central tetrahydrobenzazepine. The authors reported a 28% difference in striatal binding values between zQ175 (HD animal model) and wildtype mice at 6 months of age, increasing further to 34% three months later. Interestingly, the diseased animals expressed less D_1_ receptors in the cortex and hippocampus than their healthy counterparts, hence expanding the potential of D_1_ PET imaging in HD.

**Scheme 5:**
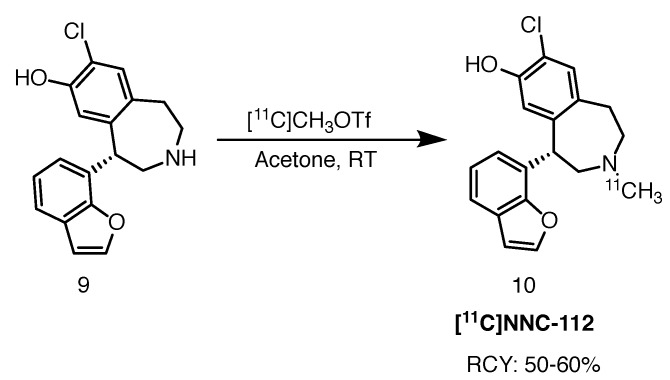
Radiolabelling of [^11^C]NNC-112, starting from the enantiomerically pure desmethyl precursor **9** [39]. RT: room temperature.

[^11^C]NNC-112 is administered in the active *S*-enantiomeric form. It is accessed in a high-yielding (50–60%, calculated from the end of bombardment) *N*-^11^C-methylation of the enantionmerically pure precursor with [^11^C]methyl triflate in acetone at room temperature (Scheme 5) [39]. Molar activity was established at 110 GBq/μmol and RCP at more than 99%.

#### 2.3.3. [^11^C]Raclopride

[^11^C]Raclopride is often used in conjunction with [^11^C]SCH-23390 to get a more in-depth image of the dopaminergic system. It is a well known antagonist of D_2/3_ receptors, used clinically, for instance, in parkinsonian patients [40].

**Scheme 6:**
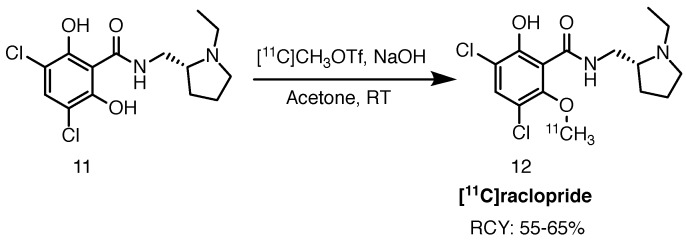
Radiolabelling of [^11^C]raclopride, starting from the desmethyl precursor **11**, published by Langer and co-workers. RCY is based on [^11^C]methyl triflate [42]. RT: room temperature.

Ehrin et al. were the first to publish the radiolabelling protocol of [^11^C]raclopride in 1985 [41]. The precursor was *N*-[^11^C]ethylated with [^11^C]ethyl iodide in the presence of 2,2,6,6-tetramethylpiperidine in DMF at room temperature. Presently, the tracer is produced routinely for clinical purposes by *O*-methylation of the hydroxyl group with [^11^C]methyl iodide or [^11^C]methyl triflate ([^11^C]CH_3_OTf) in the presence of an inorganic base. Langer and co-workers reported RCYs in the range of 55–65% and molar activities of 56–74 GBq/μmol [42]. Several groups published various optimisations to the protocol ever since, including base-free synthesis, microfluidics and captive solvent methods, among many others [43,44,45]. A typical [^11^C]raclopride synthesis is shown in Scheme 6 below.

Antonini and co-workers examined 8 symptomatic and 10 asymptomatic HD mutation carriers with [^11^C]raclopride PET, along with a 10-member healthy control group, in an effort to verify the correlation between CAG repeat lengths and striatal degeneration and age [46]. The authors unveiled a positive relationship between the two. Pavese et al. recruited 27 HD gene carriers with a minimum of 39 CAG repeats—16 symptomatic and 11 asymptomatic—for D_2_ receptor studies with [^11^C]raclopride PET [47]. Cortical reductions in D_2/3_ binding were analysed in conjunction with neuropsychological tests, such as verbal fluency, Rivermead Behavioural Memory and Boston Naming Tests. Manifest HD subjects with decreased cortical binding of the radioligand scored lower on the tests than those with unimpaired cortical dopaminergic system.

**Figure 1:**
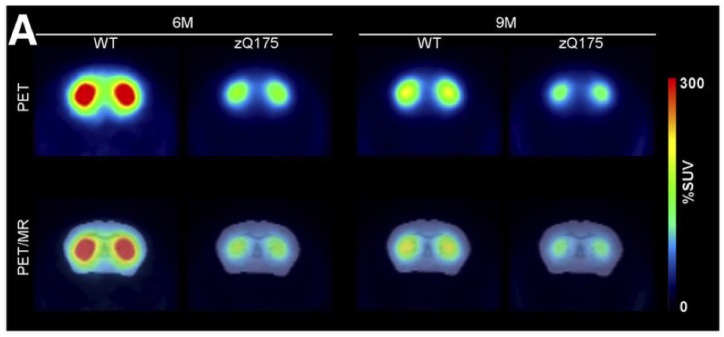
Average %SUV images of mice at 6 and 9 months of age acquired after [^11^C]raclopride injection. Lower receptor binding was observed for zQ175 mice at both timepoints. Top row: Averaged PET measurement, 15–63 min post-injection. Bottom row: PET co-registered to template MRI. Reprinted from Häggkvist and colleagues [38].

[^11^C]Raclopride was also employed in a longitudinal cross-sectional HD biomarker study in zQ175 mice by Häggkvist and colleagues, in order to pinpoint the most powerful PET tracer to detect subtle receptor changes [38]. This mouse model was engineered to express motor and cognitive impairment, as well as decreased body weight with disease progression. There was a pronounced difference in tracer uptake between heterozygous and WT animals at both 6 and 9 months of age (Figure 1). For the earlier timepoint, this difference was 40%, then increasing further to 44% at 9 months of age. In addition, both genotypes exhibited a decline in receptor density due to age progression, a natural factor.

### 2.4. GABA Receptors

Alterations of GABA, or *gamma*-amino butyric acid, receptor expression has also been studied in the context of HD. GABAergic neurotransmission is severely impaired in HD - GABAergic striatal medium spiny projection neurons are particularly targeted during the course of the disease [48,49].

#### 2.4.1. [^11^C]Flumazenil

Künig et al. studied changes in GABA receptor density in 23 HD gene carriers (10 manifest and 13 premanifest) using the PET tracer [^11^C]flumazenil, a potent benzodiazepine-based GABA antagonist [50]. The authors reported reduced [^11^C]flumazenil binding in the caudate nucleus, corresponding to the loss of projection neurons and consequently, GABA receptors. Interestingly, there was no difference in tracer binding in the putamen of the symptomatic HD subjects and healthy controls. It was proposed that an upregulatory GABA compensation mechanism was initiated, which was absent in the premanifest cohort, where neuronal loss was much less pronounced. The study was accompanied by the use of a D_2/3_ receptor radioligand, [^11^C]raclopride (for more about this tracer, see Section 2.3.3), and a glucose metabolism biomarker, [^18^F]FDG. In summary, it was reported that reduced binding of these two had taken place before that of [^11^C]flumazenil, and that this was only evident in manifest HD gene carriers.

**Scheme 7:**
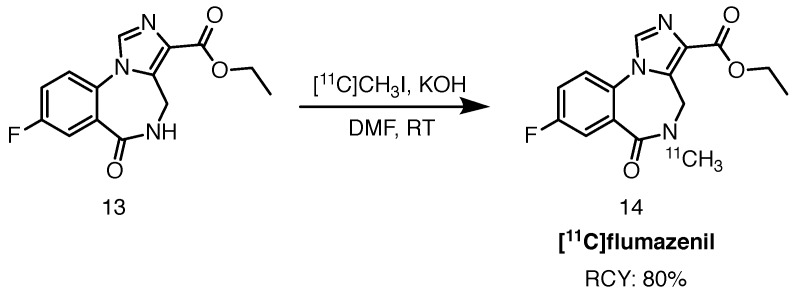
Radiolabelling of [^11^C]flumazenil, starting from the desmethyl precursor **13**, described by Cleij and colleagues [51]. A solution of the precursor in DMF is exposed to [^11^C]methyl iodide in a stream of helium, in a ‘micro-reactor’ column.

An efficient and rapid [^11^C]flumazenil radiolabelling protocol was published by Cleij et al. [51]. The typical ^11^C-methylation, achieved by trapping [^11^C]methyl iodide in a solution of the precursor, was replaced by a captive solvent method. The precursor solution was first adsorbed onto a stainless steel-packed support (inside an empty HPLC guard column), through which gaseous [^11^C]methyl iodide was subsequently fed. The precursor solution was hence efficiently dispersed, owing to the large surface area of the metallic powder. Using only 40 μg of the precursor, the authors were able to obtain the desired product with RCYs reaching 80%. Trapping efficiency of [^11^C]methyl iodide reached 90%. Very high molar activities were reported, amounting to 600 GBq/μmol, typically hard to achieve with the [^11^C]CO_2_ synthon. Due to the small size of this improvised “reactor” and reaction mixture volumes, HPLC purification was shortened significantly, arriving at an injectable solution of [^11^C]flumazenil in 20 min, counted from the end of bombardment of the cyclotron target. The synthetic route towards the tracer is shown in Scheme 7.

### 2.5. Glucose Metabolism

A great deal of publications have been devoted to the study of brain metabolism in the context of HD. Metabolic glucose changes can be traced with the use of [^18^F]FDG PET imaging. The last decade has witnessed major developments in the radiosynthetic route towards this PET tracer with regard to cassette-based synthesis using automated modules produced by companies such as Trasis and General Electric. This has enabled research institutions and hospitals to produce it in quantitative radiochemical yields and activities allowing for scanning dozens of patients per batch. In this review, however, we decided to focus on novel radiopharmaceuticals in order to highlight great advancements which have been made in the field of PET radiochemistry and the ever-expanding pool of the highly specialised molecular arsenal for HD.

### 2.6. Glutamatergic Receptors

Although the role of glutamate receptors in HD has been highlighted in numerous publications, only a few studies with glutamate-targeting PET tracers have been reported [52]. Ribeiro et al. demonstrated altered metabotropic glutamate receptor (mGluR) signalling, particularly that of group I, in presymptomatic HD mice [53]. mGluRs are members of the G protein coupled receptor family, receiving signal from glutamate, one of the main neurotransmitters. mGluR5, a member of the group I mGluR subset, is highly expressed in striatal MSNs [54].

#### 2.6.1. [^11^C]ABP-688

**Scheme 8:**
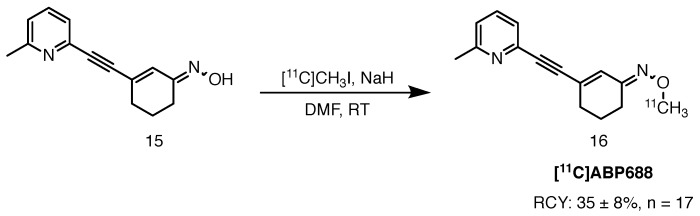
Radiolabelling of [^11^C]ABP-688, starting from the desmethyl precursor **15**, described by Amatamey and colleagues [55].

[^11^C]ABP-688 is a high affinity oxime-based structural analogue of the prototypic mGluR5 antagonist MPEP. Ametamey et al. described radiolabelling and preclinical evaluation of the tracer in 2006, followed by healthy volunteer studies in 2007 [55,56]. [^11^C]ABP-688 was accessed via ^11^C-methylation of the sodium salt of the desmethyl precursor with [^11^C]methyl iodide at 90 ${}^{\circ }$C in DMF. The tracer was obtained in 35 ± 8% RCY and RCP greater than 95% (Scheme 8). Molar activity values ranged from 100 to 200 GBq/μmol. The authors also performed an analogous investigation with the ligand labelled with an NMR active carbon-13 nuclide. Not only was formation of the *O*-methylated product confirmed, but also the *E/Z* isomeric ratio was established. It was demonstrated that a high ratio of 10:1 favouring the more potent (*E*)-[^11^C]ABP-688 can be achieved by deprotonating the precursor with sodium hydride, followed by treatment with [^11^C]methyl iodide. An alternative method involves trapping of the more reactive methylating agent, [^11^C]methyl triflate, in an acetone solution of the precursor with sodium hydroxide at room temperature. Average RCY of 25 ± 5%, at the end of synthesis, and molar activity values of 148 ± 56 GBq/μmol were obtained [57].

Bertoglio and co-workers employed [^11^C]ABP-688 in a longitudinal investigation of mGlu5 receptor changes in the Q175 mouse model of HD using PET [58]. The choice of their study was motivated by the potential of mGluR5 targeting to increase cognitive performance in an effort to find a disease-modifying treatment for HD. Animals were scanned at 6, 9 and 13 months of age. A clear loss of signal was reported in the striatum and cortex of the heterozygous mice in comparison to the wildtypes. The Q175 mice displayed a significant reduction in the nondisplaceable binding potential (BP_ND_) values between 6 and 13 months of age. The authors were cautious about drawing preliminary conclusions about the therapeutic potential of mGluR5 targeting, due to the dual role mGluR5 signalling is thought to play—neuronal activation or toxicity. In addition, they highlighted the importance of investigating age-related decline of mGluR5 availability as well as circadian-related variations. Further investigation is required to gain a better understanding of this intricate mechanism.

### 2.7. Microglia

Activation of microglia in neurodegenerative diseases has been described by various groups, yet the exact role of this mechanism is still unclear [59]. Microglia are another crucial component of the CNS, accounting for 5–15% of the entire cellular content [60]. They are heavily involved in neuroimmunity and one of their key roles is to maintain homeostasis and protect the system against pathogenesis [61]. They could also act as a trigger of damage as well as initiating compensatory action against mHTT damage. Imaging of microglia activation in HD can be performed using PET tracers binding to the 18-kDa translocator protein (TSPO). This transmembrane domain protein, formerly known as the peripheral benzodiazepine receptor (PBR), has a modest expression pattern in a healthy brain, in contrast to peripheral organs such as kidneys, heart and lungs. Increased TSPO expression has been reported in various neurodegenerative disorders, such as AD, PD and HD [60]. Consequently, the transformation from the quiescent to the activated state of the microglia has been considered a potential biomarker of HD using TSPO ligands, such as [^11^C]PK11195.

#### 2.7.1. [^11^C]PK11195

**Scheme 9:**
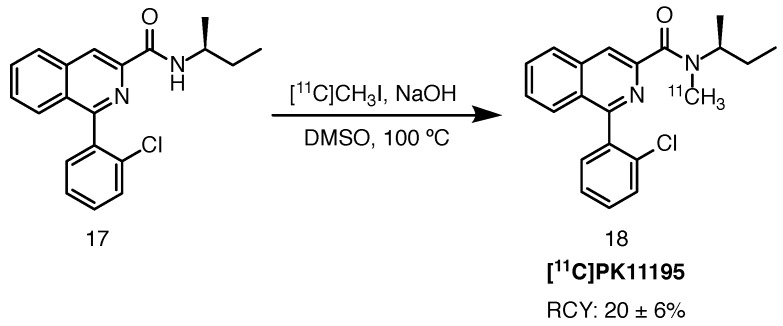
Radiolabelling of [^11^C]PK11195, starting from the desmethyl precursor **17** [62].

[^11^C]PK11195 is a first generation PET ligand targeting the TSPO protein. Radiolabelling, described by Toyama et al. in 2008, was achieved with [^11^C]methyl iodide and sodium hydroxide in DMSO at 100 ${}^{\circ }$C [62]. RCYs of 20 ± 6%, with high RCP (over 98%) and moderate molar activity (68.2 ± 18.1 GBq/μmol) at the end of synthesis were obtained (Scheme 9). In 2015, Alves and co-workers suggested an optimised radiolabelling protocol in which the tracer was performed using a captive solvent method, in an HPLC injection loop, with [^11^C]methyl iodide at room temperature [63].

The tracer was employed in a few studies with HD patients. Tai et al. recruited presymptomatic HD gene carriers who underwent [^11^C]PK11195 and [^11^C]raclopride scans [64]. Striatal and cortical binding values for the premanifest subjects were higher than those of the healthy volunteers. The results pointed towards early stage microglia activation in the course of HD progression, although no prognostic conclusion could be drawn about the age of symptom onset and [^11^C]PK11195 binding. The researchers supplemented this study with follow-up multimodal imaging with PET and MRI in a group of manifest HD individuals [59]. The results were divided regionally into brain regions of HD manifestations (motor, cognitive and psychiatric) and levels of microglia activation, D_2_ receptor and neuronal loss (measured as decreases in volume using MRI volume-of-interest analysis) for each of the subject groups (normal brain, premanifest and manifest HD brains). An increase in radioligand binding was observed in the sensorimotor striatum, globus pallidus, substantia nigra and red nucleus, all the brain areas involved in motor functioning. Pathological changes in microglia activation were reported for the first two regions for the premanifest and manifest subjects. The same uptake pattern of the tracer was found in brain regions responsible for cognitive and psychiatric functions (e.g., associative striatum, insula, amygdala, hypothalamus). These results provide further evidence that activated microglia could orchestrate crucial mechanisms behind neuronal loss in HD, although the precise mode of action is not yet well understood.

#### 2.7.2. [^18^F]PBR06

In 2018, this radioligand was recognised by Simmons et al. as a potential biomarker for monitoring the therapeutic effect of an inflammation reducing agent LM11A-31 in a mouse model of HD [65]. [^18^F]PBR06, a second generation TSPO ligand, was previously investigated in humans and non-human primates by Imaizumi et al. and Fujimura et al. [66,67]. Simmons highlighted the advantage of this [^18^F]fluorinated tracer over [^11^C]PK11195 with respect to half-life, affinity for the TSPO protein and better signal-to-noise ratio. The authors used PET imaging after administration of LM11A-31 to R6/2 and BACHD mice, both expressing mutant huntingtin protein. The vehicle-treated transgenic animals exhibited higher uptake of the tracer in the striatum, cortex and hippocampus, compared to wildtypes and LM11A-31 treated counterparts. These findings correlate with data acquired with [^11^C]PK11195 in manifest and premanifest HD subjects.

**Scheme 10:**
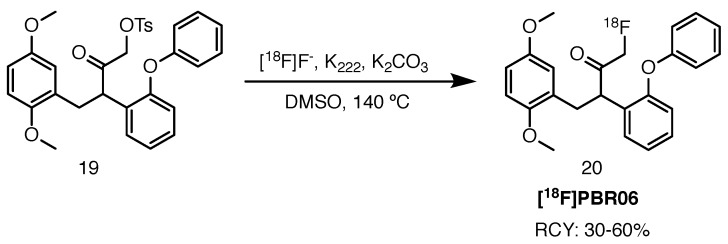
Radiolabelling of [^18^F]PBR06, starting from the tosyloxy precursor **19** [68].

Synthesis of [^18^F]PBR06, reported by Wang et al. in 2011, was achieved on an in-house automatic synthesis module by heating the corresponding tosyloxy precursor in the presence of potassium carbonate and Kryptofix 222 in DMSO at 140 ${}^{\circ }$C (Scheme 10) [68]. RCYs ranged from 30% to 60%, with molar activity values reaching 222 GBq/μmol. This route offers an improvement with regards to previously reported protocols by Briard et al., who performed radiolabelling with the bromo precursor, achieving lower RCYs and molar activity values [69]. Inferior performance of the latter can be attributed to the worse leaving group character of the bromide, in addition to problematic separation of the tracer from precursor using high-performance liquid chromatography.

### 2.8. Phosphodiesterase 10A

Phosphodiesterase 10A (PDE10A), a member of phosphodiesterases (PDEs), is an enzyme involved in deactivation of cyclic adenosine monophosphate and cyclic guanosine monophosphate (cAMP and cGMP, respectively). It is highly expressed in MSNs, where it regulates their excitability [70]. As mentioned earlier, MSNs are severely deregulated in various neurodegenerative pathologies, including HD. Hebb et al. reported that reduction of striatal PDE10A expression in transgenic R6/1 and R6/2 mice models of HD preceded motor impairment [71]. Several PET tracers targeting the enzyme have been proposed and studied preclinically and in humans. They were described by Boscutti et al. in their exhaustive review article in 2019 [72].

#### 2.8.1. [^18^F]JNJ42249152

**Figure 2:**
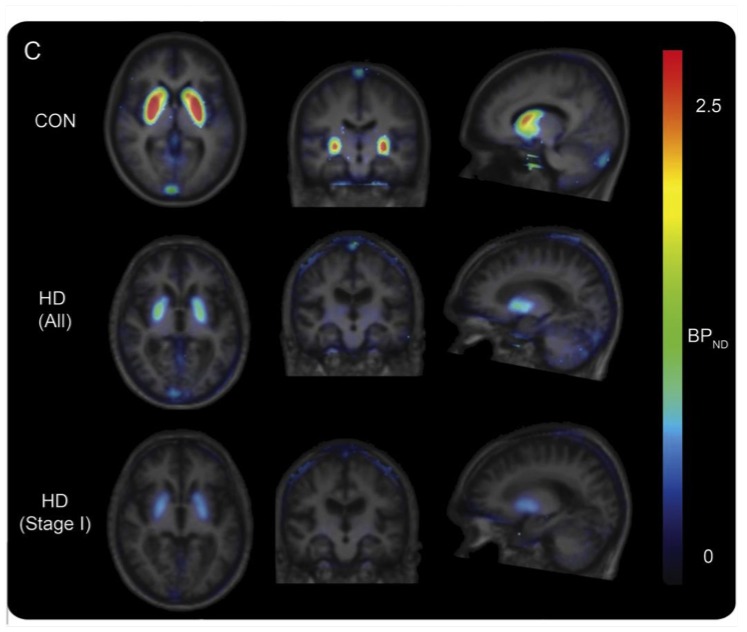
Mean PDE10A BP_ND_ maps, obtained with [^18^F]JNJ-42249152, projected on the mean group T1 MRI for 11 healthy controls, 5 manifest and 2 premanifest HD participants (stage I). Partial volume correction was performed. Loss of striatal PDE10A is clearly visualised in HD patients using this ligand. Reprinted from Ahmad and colleagues [76].

**Scheme 11:**
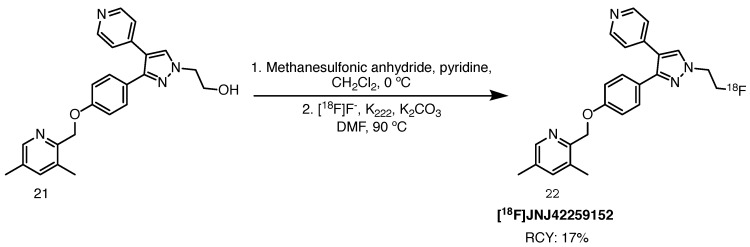
Most efficient radiolabelling of [^18^F]JNJ42249152, starting from the hydroxy building block **21**. The precursor for labelling, the mesylate derivative of compound **21**, is unstable, hence it must be produced shortly before the reaction. It is introduced into a reactor vial with dried [^18^F]F^−^ after solid phase extraction the day before [73].

Radiolabelling of this PDE10A inhibitor was first described by Andrés et al. in 2011 (Scheme 11) [73]. The precursor was synthesised through an 8-step route starting from 4-hydroxybenzoic acid methyl ester. Stability of the *O*-mesyl protected precursor proved to be a bottleneck in the process. Although isolation of the compound was possible, optimum results were reached when the precursor was synthesised the day before, having been passed through a Sep-Pak C18 cartridge and dried in vacuo overnight. It was then introduced to [^18^F]fluoride and [^18^F]fluorination proceeded with 17% RCY (based on [^18^F]F^−^, *n* = 8), with RCP higher than 97% after reverse-phase HPLC purification. The average molar activity (*n* = 8) was established at 167 GBq/μmol. In vitro PDE10A inhibition assays provided favourable pIC_50_ and lipophilicity (clogP) values of 8.8 and 3.66, respectively, suggesting a potent ligand capable of crossing the blood–brain barrier. The authors also performed biodistribution studies in male Wistar rats and observed a time-dependent increased striatal accumulation and slow 2-to-30 min washout, compared to the hippocampus, cortex and cerebellum, in line with PDE10A expression levels. The evaluation of [^18^F]JNJ42249152 as a PET ligand for PDE10A imaging continued with in-human studies performed by Van Laere and co-workers in collaboration with Janssen Pharmaceuticals [74,75]. Six healthy male volunteers were subjected to a whole body PET/CT scan. Unsurprisingly, the radiopharmaceutical exhibited rapid uptake in the striatum, particularly the putamen, reaching its peak at 12–15 min, and a high clearance rate. The test-retest evaluation in a concomitant human study with healthy male subjects was characterised by low intra-subject variability. The presence of two plasma metabolites, resulting from the cleavage of the 3,5-dimethylpyridine motif and yielding the radioactive phenol, is one of the limitations of this tracer. Ahmad and co-workers also tested it in an additional cohort of 5 HD sufferers, determined to unveil the fate of PDE10A in the presence of mHTT [76]. This quest was fuelled by contradictory data about enzyme depletion in a post-mortem striatal analysis of HD-affected individuals versus overexpression in a mouse model of the disease. Results of this in vivo experiment provided further evidence towards a reduction of phosphodiesterase 10 A in the presence of mHTT, however no clear link to clinical rating scales was demonstrated (Figure 2). The authors also presented alternative scenarios, according to which PDE10A levels were correlated with dropping cAMP levels.

#### 2.8.2. [^18^F]MNI-659

[^18^F]MNI-659 and its derivative [^18^F]MNI-654 were proposed as promising PDE10A radioligands by Barret et al. in 2012 [77]. Studies in non-human primates exhibited the highest update in the putamen and globus pallidus. Cerebellum, with lowest tracer accumulation, was used as a reference region for BP_ND_ quantification. In their follow-up investigation in humans, [^18^F]MNI-654 was disregarded as a potential PDE10A tracer due to slow kinetics. Its analogue, however, displayed a highly favourable in vivo profile.

**Scheme 12:**
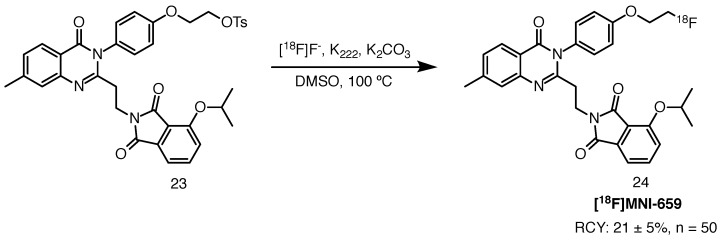
Radiolabelling of [^18^F]MNI-659, starting from the tosylate precursor **23** [77].

[^18^F]MNI-659 was accessed via S_N_2 substitution of the tosylate leaving group with [^18^F]fluoride, in the presence of Kryptofix 222 and potassium carbonate. The reaction was performed in DMF at 100 ${}^{\circ }$C using a TRACERlab FX_FN_ (GE Healthcare) automated unit. Reverse-phase HPLC purification on a C18 column yielded the injectable solution of the radioligand in 21 ± 5% RCY (*n* = 50). RCP was over 99%, with molar activity values exceeding 185 GBq/μmol. The reaction is shown in Scheme 12.

Plasma analysis revealed a moderate metabolic profile of the tracer, with 20% intact compound at 2 h post-injection. The presence of radioactive metabolites did not raise concern as they were more polar than the tracer itself, hence unlikely to cross the blood–brain barrier. The highest [^18^F]MNI-659 uptake was recorded in the putamen, globus pallidus and caudate, in line with PDE10A expression patterns. Peak accumulation in these bran regions was observed 10–20 min post-injection, while cerebellum exhibited much faster washout, making it a suitable candidate for the reference region (to assess non-specific binding).

Russell et al. evaluated the potential of [^18^F]MNI-659 as a PET tracer for HD imaging [78]. The cohort included nine healthy volunteers and 11 HD sufferers, of which three were ranked as premanifest and the remaining eight as manifest. PET images revealed a clear lack of striatal PDE10A binding among HD-affected individuals that correlated with disease severity. The same group published a follow-up study two years later [79]. Along with the healthy controls, eight HD patients were recruited, 2 premanifest and 6 manifest with early stage disease rating. The subjects were scanned twice, with a one year difference in between. Loss of radioligand binding in the putamen, caudate nucleus and globus pallidus was pronounced among the HD patients, 16.6%, 6.9% and 5.8%, respectively, with only 1% decline for the healthy volunteers.

#### 2.8.3. [^11^C]IMA-107

Another potential PET tracer for the imaging of HD was developed in collaboration with Imanova (now Invicro). A series a potent PDE10A inhibitors was proposed and characterised in pigs, baboons and humans [80]. The ligands all contained a central pyrazolo[1,5-*a*]pyrimidine unit. The choice was narrowed down to three tracers after the results of the in vivo evaluation in pigs, where [^11^C]IMA-107 displayed specific binding to PDE10A during a blocking study. The highest uptake was noted in the striatum and kinetics were suitable for PET application. The next step involved testing the radioligands in baboons, where kinetics were generally much slower than in the porcine brain, yet [^11^C]IMA-107 proved to be the most specific tracer in the series. Studies in healthy humans showed a similar uptake profile, with the highest standardised uptake values (SUVs) for the putamen and globus pallidus. Reversible kinetics and favourable washout confirmed its potential as a PET tracer for the imaging of PDE10A alterations.

**Scheme 13:**
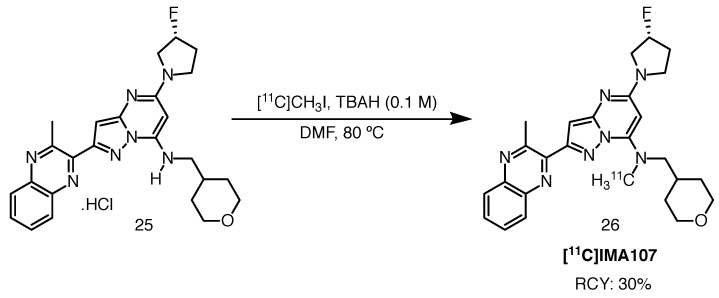
Radiolabelling of [^11^C]IMA-107, starting from precursor **25** [80].

Radiosynthesis of the tracer, also reported by Plisson et al., proceeded with an estimated RCY of 30% and the tracer can be produced in clinically useful quantities. [^11^C]methylation of the amine moiety with [^11^C]methyl iodide was achieved in the presence of tetrabutylammonium hydroxide in DMF at 80 ${}^{\circ }$C (Scheme 13). The total synthesis time, from the end of cyclotron bombardment to obtain [^11^C]carbon dioxide, and arriving at the formulated injectable product, was approximately 40 min. RCY was approximately 30% and RCP exceeded 95%. Moderate to good molar activity values for human studies were obtained, 62–287 GBq/μmol. Radiolabelling with carbon-11 is particularly beneficial during preclinical development, where a higher throughput can be achieved due to its short half-life and consequently, the possibility to use automated modules to produce ^11^C-labelled radioligands more than once within a day. Despite the presence of a fluorine atom, radiolabelling of the scaffold with fluorine-18 was not attempted.

Niccolini et al. assessed the discriminatory power of this radioligand for the imaging of PDE10A alterations in 12 early premanifest carriers of the *mHTT* mutation [81]. Individuals were chosen such that the symptom onset would not appear earlier than 25 ± 6.9 years with 90% probability. The resulting PET data were compared to those of the healthy controls. Interestingly, the tracer was able to detect differences in PDE10A expression years before the appearance of the first clinical symptoms of HD. Binding was reduced in the striatum and globus pallidus, with the opposite effect in the motor thalamic nuclei and similar uptake in substantia nigra and ventral striatum. Changes of phosphodiesterase 10A expression affected the dorsolateral striatum primarily, sparing the limbic and cognitive parts. The authors speculated that the increased [^11^C]IMA-107 binding in motor thalamic structures may be a compensatory mechanism, which eventually collapses, ending in symptom manifestation.

### 2.9. Synaptic Vesicle Protein 2A

Synaptic vesicles play a crucial yet cryptic part of the intricate neurotransmission process. Synaptic vesicle glycoprotein 2A, or SV2A, is expressed extensively in synaptic vesicles of the central nervous system [82]. Levetiracetam, also known by its brand name Keppra, is an anti-epileptic drug which binds to SV2A and impedes the action of voltage-dependent Ca^2+^ channels, hence reducing neurotransmission [83]. Defective SV2A expression has been reported in various neuropathologies, such as Alzheimer’s disease and pertinent to this review, HD. Several research articles highlighted the potential use of SV2A as a biomarker of synaptopathies with PET [82,84].

#### 2.9.1. [^11^C]UCB-J

[^11^C]UCB-J is a more structurally complex derivative of levetiracetam, with a central pyrrolidinone motif. After a thorough screening, UCB Pharma proposed three potential SV2A tracers, which exhibited favourable in vitro pharmacokinetics and were predicted to cross the blood-brain barrier [84,85].

**Figure 3:**
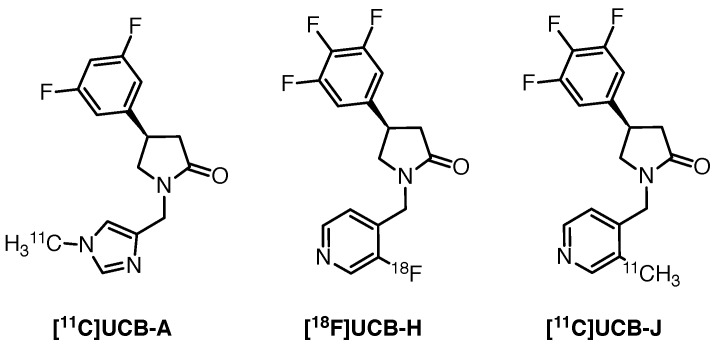
Radioligands for SV2A imaging proposed by UCB Pharma [84].

[^11^C]UCB-A (Figure 3, left) evaluation in six epileptic individuals and two healthy controls revealed slow plasma and brain kinetics, a challenge for kinetic modelling. [^18^F]UCB-H (Figure 3, middle) performed better in rats and non-human primates. Further clinical evaluation of [^18^F]UCB-H was positive—the tracer accumulated readily in all relevant brain regions. The lack of an accurate reference region imposed the need for invasive arterial blood sampling in order to allow accurate quantification, however, an image-derived input function was later proposed by Bahri and co-workers as a good estimate of the arterial input function [86]. [^11^C]UCB-J (Figure 3, right) outperformed [^11^C]UCB-A and [^18^F]UCB-H in non-human primate and rat studies. It exhibited high uptake and very fast kinetics, allowing convenient scanning, with the short half-life of carbon-11 in mind. Clinically, the tracer accumulated rapidly in the brain, following the SV2A expression pattern, with washout starting approximately 20 min post-injection.

**Scheme 14:**
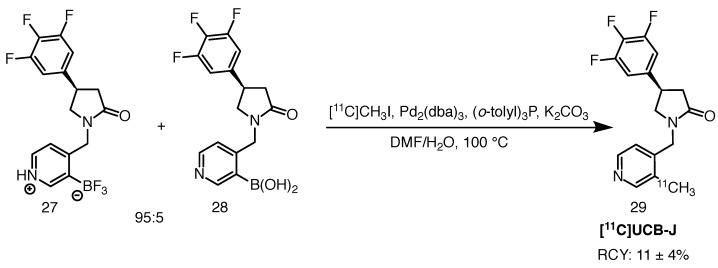
Radiolabelling of [^11^C]UCB-J, starting from a 95:5 mix of precursors **27** and **28** [84,87].

Radiolabelling of [^11^C]UCB-J proceeds via palladium-mediated coupling of the trifluoroborate precursor with [^11^C]methyl iodide, also known as Suzuki-Miyaura coupling (Scheme 14). The precursor is usually enriched with 2–5% of the boronic acid counterpart in order to ensure efficient radiolabelling, although the exact need for this is not entirely understood [84,87]. Reaction success is dependent on the purity of the precursor as well as proper handling of the Pd${}_{2}$(dba)${}_{3}$ catalyst and degassing the reaction mixture with inert gas prior to use. Nabulsi et al. reported 11 ± 4% RCY and molar activities reaching over 566.1 GBq/μmol [87]. The scheme is presented below.

Recently, DiFilippo and co-workers published an improved protocol for [^11^C]UCB-J synthesis, having struggled to reproduce the yields provided by Nabulsi and colleagues [88]. The authors introduced a hydrolysis step of the precursor prior to ^11^C-methylation, in order to generate the corresponding boronic acid, which was then dissolved in DMF and exposed to [^11^C]methyl iodide. The authors then followed the protocol of Nabulsi et al., except for the temperature—they heated the reaction mixture at 135 ${}^{\circ }$C for 10 min. [^11^C]UCB-J was obtained in 56 ± 7% RCY, with RCP exceeding 99% and a high molar activity of 477.3 ± 133.2 GBq/μmol.

To the best of our knowledge, Bertoglio and co-workers were the first ones to employ [^11^C]UCB-J in a study with a HD animal model [89]. The group described kinetic modelling of the radioligand in 8-month-old Q175DN and wildtype mice. The tracer was synthesised on an automated module unit (Comecer) using the previously described protocol with a 95:5 mixture of the trifluoroborate and boronic acid precursors (Scheme 14). No RCYs were reported, but RCP over 99% and moderate molar activity of 78 ± 23 GBq/μmol were obtained. Along with baseline scans, a blocking study with leviteracetam was performed. The authors reported dose-dependent blocking of the radioligand, in line with SV2A expression. Relatively low non-specific binding was revealed. This work serves as premise for further evaluation of the tracer in different HD animal models, with the use of non-invasive quantification based on the one tissue compartment model (1TCM) and an image-derived input function.

## 3. Conclusions

Alterations in expressions of adenosine A_1_ and A_2_, cannabinoid CB_1_, dopaminergic D_1_ and D_2_ and glutamatergic mGluR5 receptors, together with activation of microglia, PDE10A and SV2A protein dysfunction have been considered as promising biomarkers of HD. Advanced PET imaging using potent and specific radioligands tagged with carbon-11 or fluorine-18 enable visualisation of biochemical changes and intricate mechanisms related to these targets. As of now, no single PET tracer binding to mutant huntingtin protein has been reported. None of the molecular targets presented in this review can act as a stand-alone tool for HD progression monitoring, but the knowledge gathered since the mapping of the mHTT mutation in 1993, has brought researchers even closer to understanding the pathology and eventually, finding the highly sought after disease-modifying treatment.

## Abbreviations

The following abbreviations are used in this manuscript:

A_1_ and A_2A_	Adenosine receptors
AD	Alzheimer’s disease
BP_ND_	Binding potential
Bq	Becquerel
CAG	Cytosine-adenine-guanine
CB_1_	Cannabinoid receptor type 1
CNS	Central nervous system
D_1_, D_2_ and D_3_	Dopamine receptors
DMF	Dimethyl formamide
DMSO	Dimethyl sulfoxide
GABA	*gamma*-Aminobutyric acid
GMP	Good manufacturing practice
HD	Huntington’s disease
HPLC	High performance liquid chromatography
IB	Inclusion body
K_222_	Kryptofix 222
MeCN	Acetonitrile
mHTT	Mutant huntingtin
MRI	Magnetic Resonance Imaging
MSN	Medium spiny neuron
OTf	Trifluoromethanesulfonate, triflate
PET	Positron Emission Tomography
PD	Parkinson’s disease
RCP	Radiochemical purity; defined as the absence of other radiochemical compounds/species
RCY	Radiochemical yield; defined as the amount of activity in the product expressed as the percentage (%) of starting activity used in the considered process, all RCYs presented in this review are decay corrected
RT	Room temperature
SUV	Standardised uptake value
WT	Wildtype
